# The Effect of Excessive Sulfate in the Li-Ion Battery Leachate on the Properties of Resynthesized Li[Ni_1/3_Co_1/3_Mn_1/3_]O_2_

**DOI:** 10.3390/ma14216672

**Published:** 2021-11-05

**Authors:** Jimin Lee, Sanghyuk Park, Mincheol Beak, Sang Ryul Park, Ah Reum Lee, Suk Hyun Byun, Junho Song, Jeong Soo Sohn, Kyungjung Kwon

**Affiliations:** 1 Department of Energy and Mineral Resources Engineering, Sejong University, 209 Neungdong-ro, Gwangjin-gu, Seoul 05006, Korea; txoio7612@naver.com (J.L.); shpark@sejong.ac.kr (S.P.); doqmsalscjf@naver.com (M.B.); 2 SungEel HiTech Co., Ltd., 143-12, Gunsansandan-ro, Gunsan-si 54002, Korea; qkrtkdfuf68@sungeel.com (S.R.P.); ahreum@sungeel.com (A.R.L.); shbn14@sungeel.com (S.H.B.); 3 Korea Electronics Technology Institute, 25 Saenari-ro, Bundang-gu, Seongnam-si 13509, Korea; 4 Mineral Resources Research Division, Resources Recovery Research Center, Korea Institute of Geoscience and Mineral Resources, 124, Gwahak-ro Yuseong-gu, Daejeon 34132, Korea

**Keywords:** Li-ion battery, cathode material, sulfate, impurity, leachate

## Abstract

In order to examine the effect of excessive sulfate in the leachate of spent Li-ion batteries (LIBs), LiNi_1/3_Co_1/3_Mn_1/3_O_2_ (pristine NCM) and sulfate-containing LiNi_1/3_Co_1/3_Mn_1/3_O_2_ (NCMS) are prepared by a co-precipitation method. The crystal structures, morphology, surface species, and electrochemical performances of both cathode active materials are studied by scanning electron microscopy (SEM), X-ray diffraction (XRD), X-ray photoelectron spectroscopy (XPS), and charge-discharge tests. The XRD patterns and XPS results identify the presence of sulfate groups on the surface of NCMS. While pristine NCM exhibits a very dense surface in SEM images, NCMS has a relatively porous surface, which could be attributed to the sulfate impurities that hinder the growth of primary particles. The charge-discharge tests show that discharge capacities of NCMS at C-rates, which range from 0.1 to 5 C, are slightly decreased compared to pristine NCM. In dQ/dV plots, pristine NCM and NCMS have the same redox overvoltage regardless of discharge C-rates. The omnipresent sulfate due to the sulfuric acid leaching of spent LIBs has a minimal effect on resynthesized NCM cathode active materials as long as their precursors are adequately washed.

## 1. Introduction

Li-ion batteries (LIBs) have been extensively used in various portable electronics and electric vehicles in combination with high energy and power density [1,2]. However, tremendous amounts of end-of-life batteries have been piled up in landfill, and flammable and toxic elements in spent LIBs would cause not only fire but also soil contamination [3,4,5]. At the same time, the spent LIBs could also offer huge economic benefits by recovering valuable elements in them [6,7]. In respect of LIB composition, there are many portions of valuable metals including cobalt, nickel, and lithium. When the spent LIBs are treated properly, worthy elements such as cobalt and lithium can be regained. From the viewpoint of economic and environmental issue, the recycling of spent LIBs is essential for the present and future generation [8].

In the recycling process of batteries, the pretreatment of spent batteries, including discharge, dismantling, classification, and separation, usually precedes the hydrometallurgy-based recycling process [9]. The hydrometallurgy-based process involves a leaching step to extract desired metals from cathode materials by using leaching agents such as sulfuric acid [10]. A large number of research articles have investigated the leaching behaviors of metals in the spent LIBs, with the optimization of the leaching step leading to the maximization of leaching efficiencies for valuable metals. [11,12,13,14]. Because sulfuric acid leaching is the most popular in LIB recycling and usual metal sources for the synthesis of LIB cathode active materials are sulfate salts such as NiSO_4_, CoSO_4_, and MnSO_4_, the investigation on the effect of sulfate in pristine and resynthesized cathode active materials would be essential.

Previously, Ban et al. found that sulfur in LiNi_x_Co_y_Mn_z_O_2_ (NCM) enhanced the electrochemical performance of the cathode active materials, in which sulfur was incorporated into NCM by calcination [15]. Interestingly, discharge capacity and rate capability were improved with the small amount of sulfur doping (0.4 and 0.5 wt%), which is contrary to the common sense of LIB industry that sulfur is a harmful impurity. The authors explained the reason being that sulfur forms a Li_2_SO_4_ phase, which would provide fast diffusion channels for lithium ions on the surface. Recently, Li et al. examined the concentration gradient S-doped NCM and argued that a proper amount of sulfate stabilizes the crystal structure with good cycle performance [16]. However, these studies first prepared precursors for NCM by co-precipitation and conducted the sulfur doping by calcination, which is unlikely in the NCM resynthesis for LIB recycling, because the leachate for the subsequent co-precipitation of NCM already contains excessive sulfate. Therefore, the effect of sulfur in NCM, especially in the case of NCM resynthesis, should be investigated with S-doped NCM that is prepared by co-precipitation.

In this work, we synthesize LiNi_1/3_Co_1/3_Mn_1/3_O_2_ (pristine NCM) and sulfate-containing LiNi_1/3_Co_1/3_Mn_1/3_O_2_ (NCMS) using co-precipitation in order to investigate the effect of excessive sulfate in the leachate for the NCM resynthesis. Since an actual LIB leachate could contain various types of unidentified impurities, we prepare a simulated LIB leachate with 4 M of extra lithium sulfate as a sulfur source. Our previous report, on the effect of residual lithium in resynthesized NCM, revealed that lithium originating from lithium sulfate in a simulated leachate hardly affects the LIB performance as long as the NCM precursors are washed appropriately [17]. Thus, we could attribute the properties of NCMS, which was prepared from the simulated LIB leachate with extra lithium sulfate, to sulfate in the leachate. The structural characterization is performed by scanning electron microscopy (SEM), X-ray diffraction (XRD), and X-ray photoelectron spectroscopy (XPS). For electrochemical performance, we fabricate CR2032-type coin cells using a Li metal foil as anode to measure charge and discharge capacities at different C-rates, which range from 0.1 to 5 C.

## 2. Experimental Section

**Figure 1:**
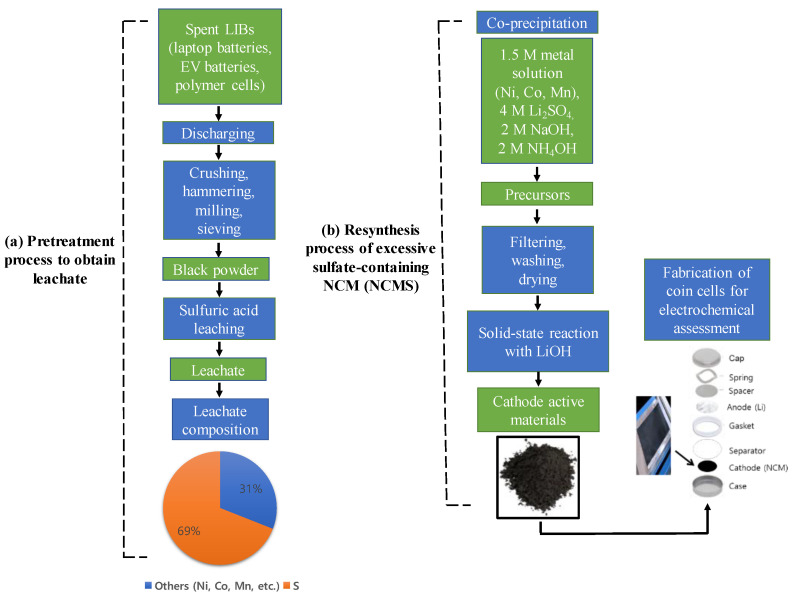
Flowchart of methodology for the resynthesis of excessive sulfate-containing NCM from spent LIBs. (**a**) Typical pretreatment process of spent LIBs with subsequent acidic leaching process using H_2_SO_4_, indicating the excessive sulfur content in leachate. (**b**) Synthesis process of sulfate-containing NCMS(OH)_2_ precursors using the co-precipitation method and NCMS cathode active materials via a solid-state reaction, followed by a fabrication of coin cells for electrochemical assessment.

Figure 1 shows the flowchart of methodology simulating a resynthesis process of excessive sulfate-containing NCM from leachate of spent LIBs, which include cylindrical 18650 laptop batteries, electric vehicle batteries, and polymer cells. Incidentally, the spent LIBs are mainly composed of graphite as anode material, and LiCoO_2_ and NCM as cathode materials.

### 2.1. Synthesis and Characterization of Materials

Ni_1/3_Co_1/3_Mn_1/3_(OH)_2_ and sulfate-containing Ni_1/3_Co_1/3_Mn_1/3_(OH)_2_ were synthesized using the co-precipitation method. The composition of actual leachate of spent LIBs from a LIB recycling company (SungEel HiTech, Gunsan-si, Korea) was considered to simulate the amount of sulfur in the actual leachate. A total of 2 M of ammonia solution as a chelating agent and 1.5 M of metal solution (NiSO_4_∙6H_2_O, CoSO_4_∙7H_2_O, and MnSO_4_∙H_2_O in 1:1:1 mole ratio with 4 M of extra Li_2_SO_4_ (99%, Alfa Aesar, Ward Hill, MA, USA)) were pumped into a continuous stirred reactor. 2 M of NaOH solution was automatically injected into the reactor by a pH-controlled pump to maintain a pH of 11.52. The reactor was kept at a temperature of 40 °C and a stirring speed of 1000 rpm for about 75 h. The resultant precursors were filtered and washed with distilled water several times and dried in an oven at 80 °C. The final cathode active materials (pristine NCM and NCMS) were prepared by calcinating a mixture of the hydroxide precursors and Li_2_CO_3_ as a lithium source at 1000 °C for 8 h under air atmosphere. In order to identify the crystal structure of the NCM and NCMS materials, an XRD technique (X’Pert, PAN analytical, Cu Kα radiation, Almelo, The Netherlands) was carried out with a step size of 0.026° in a 2θ range from 10° to 80°. The morphological characterization of the materials was performed using a field emission SEM (FE-SEM, SU-8010, Hitachi Ltd., Tokyo, Japan). XPS (K-Alpha 1063, Thermo Fisher Scientific, Waltham, MA, USA) was used to examine the presence of sulfur in the structure of NCMS.

### 2.2. Electrochemical Analysis

Electrochemical properties were investigated using CR2032-type coin cells, which were fabricated in a moisture-controlled glove box under argon atmosphere. Cathodes were prepared by mixing the cathode active materials, polyvinylidene fluoride (KF 1100) binder, and carbon black (Super-P) in a mass ratio of 95:3:2 respectively. Cells were integrated with the prepared cathodes, lithium metal as an anode, polyethene film as a separator, and 1 M LiPF_6_ in a mixture of ethyl methyl carbonate and ethylene carbonate (2:1 volume ratio) as an electrolyte. Charge-discharge tests were performed from 3.0 to 4.3 V (vs. Li/Li^+^) at room temperature.

## 3. Results and Discussion

**Figure 2:**
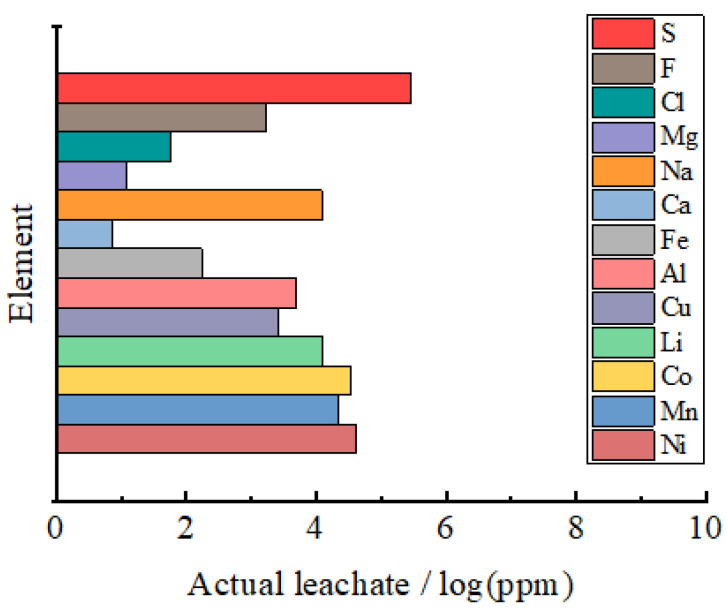
The content of various elements in the actual LIB leachate.

Figure 2 shows the content of various elements in the actual LIB leachate from a LIB recycling company (SungEel HiTech, Gunsan-si, Korea). Li, Ni, Mn, and Co are detected as major constituents of cathode materials and other metal elements, including Al, Cu, and Na, originate from current collectors and the pretreatments for LIB recycling [18,19,20]. Notably, nonmetal elements, including F, Cl, and S, are detected in the LIB leachate. Each element was analyzed by the following methods: Inductively coupled plasma for metal elements, absorptiometric analysis using La alizarin complexone for F, AgNO_3_ titration for Cl, and barium sulfate precipitation for S. F can originate from residual electrolytes such as LiPF_6_ [21], and Cl might derive from the discharging process using NaCl [22]. Since sulfuric acid is commonly used for leaching LIBs, a great amount of S is present in the leachate and S has the highest concentration among all the elements [23]. This suggests that the influence of S on the LIB performance of resynthesized cathode active materials needs to be investigated.

**Figure 3:**
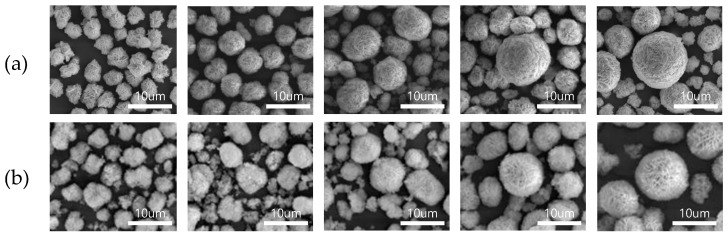
SEM images of the precursors of (**a**) pristine NCM and (**b**) NCMS according to the co-precipitation time ((**a**) 16, 24, 40, 64, and 72 h, (**b**) 5, 26, 40, 64, and 75 h).

The SEM images in Figure 3 show the morphology of the precursors of pristine NCM and NCMS according to the co-precipitation time. Both precursors have spherical secondary particles consisting of needle-like primary particles, with an average secondary particle diameter of ~10 µm. The particles grow bigger and more spherical as the co-precipitation time increases. Compared with pristine NCM, which has the continuous particle growth with decreasing particulates, NCMS shows sluggish particle growth after 64 h. Besides, NCMS has a relatively porous surface even when the co-precipitation appears to stop, while pristine NCM exhibits a very dense surface. This may attribute to the sulfate impurities that hinder the growth of primary particles.

**Figure 4:**
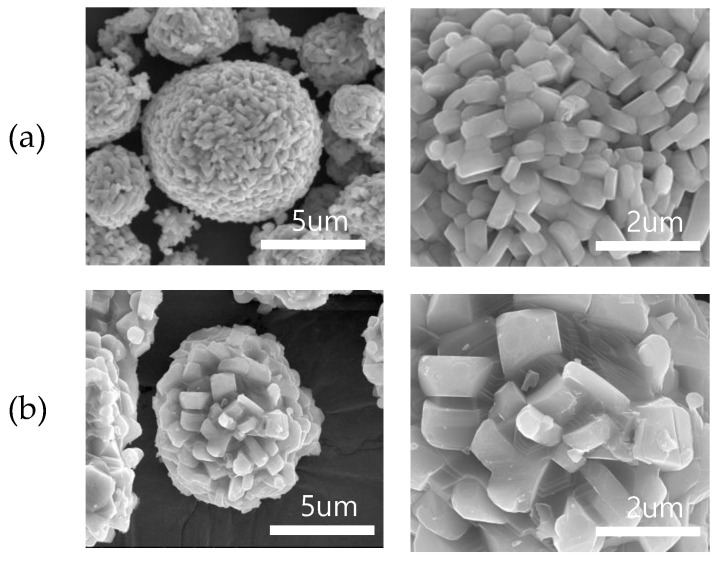
SEM images of the cathode active materials of (**a**) pristine NCM and (**b**) NCMS.

Figure 4 shows the SEM images of the cathode active materials of pristine NCM and NCMS. The difference between pristine NCM and NCMS is more distinct in the cathode active materials than their precursors. NCMS, whose precursors have porous surface, is likely to have more voids and its primary particles agglomerate more than pristine NCM during the calcination step. This may cause the structure instability and deteriorate the electrochemical performance [24]. Besides, larger primary particles decrease the lithium-ion conductivity due to their longer diffusion paths [25]. These results suggest that excessive sulfate affects the surface morphology of NCM, which could exert a noticeable influence on the LIB performance.

**Figure 5:**
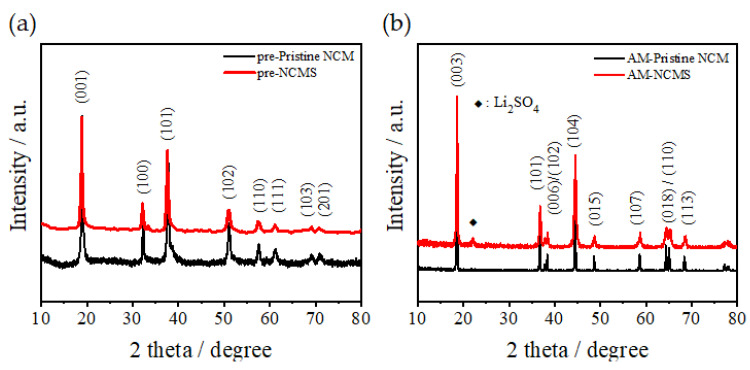
XRD patterns of the (**a**) precursors and (**b**) cathode active materials of pristine NCM and NCMS.

**Figure 6:**
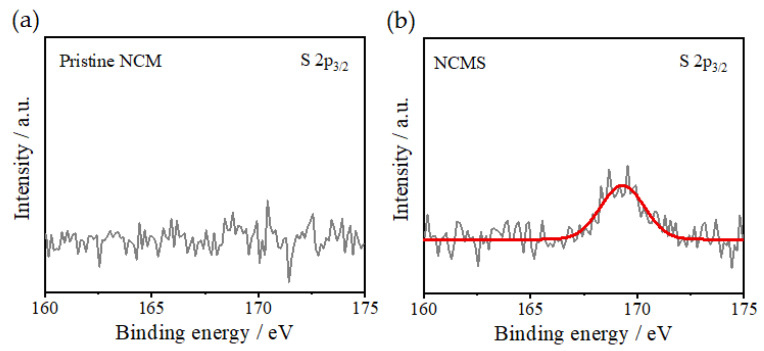
XPS profiles of the cathode active materials of (**a**) pristine NCM and (**b**) NCMS.

Figure 5a shows that all diffraction peaks are well consistent with β-Ni(OH)_2_ with no impurity phase [26]. There is no distinct difference between pristine NCM and NCMS in the precursors. Figure 5b reveals that both pristine NCM and NCMS have the well-layered structure of α-NaFeO_2_ type with the space group *R-3m* [27,28]. However, an Li_2_SO_4_ impurity phase around 22° peak is observed in NCMS. This result indicates that some sulfate impurities are still present in the precursors after filtering and washing, and these sulfate impurities appear in the cathode active materials after calcination and could lead to poor performance in a charge-discharge test. These weak peaks related to Li_2_SO_4_ phase are also proven by the presence of sulfur in the following XPS analysis (Figure 6). Figure 6b exhibits a binding energy of 169.2 eV, which is assigned to hexavalent S 2p_3/2_ of the SO_4_^2−^ groups [29]. This result identifies the presence of sulfate groups on the surface of NCMS.

**Figure 7:**
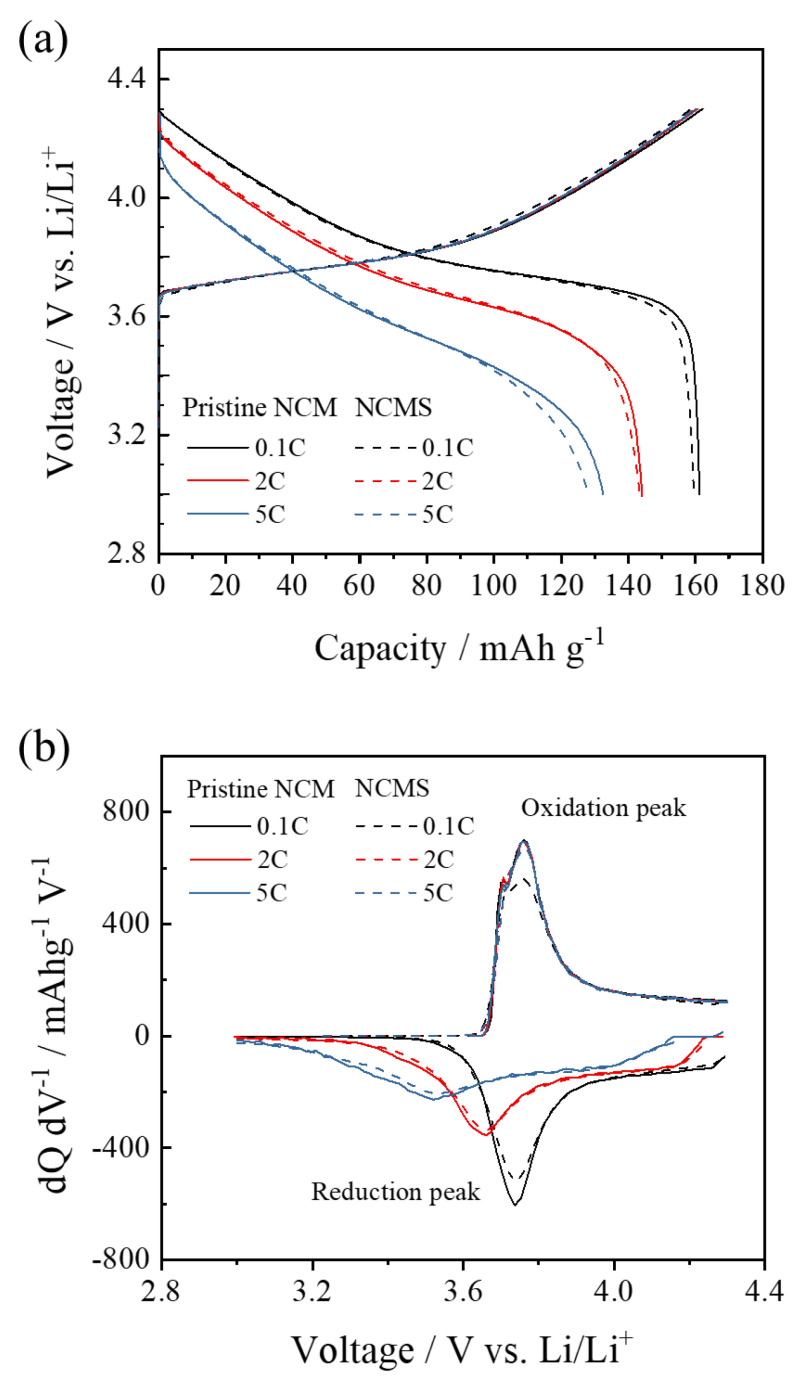
(**a**) Charge and discharge capacities and (**b**) dQ/dV plots of pristine NCM and NCMS at 0.1, 2, and 5 C.

The electrochemical performance of the charge-discharge profiles for pristine NCM and NCMS is presented in Figure 7a. For both samples, the C-rate during the charge was fixed at 0.1 C, while the C-rates during the discharge were varied from 0.1 to 5 C. Compared to pristine NCM, the discharge capacities of NCMS at each C-rate is slightly decreased. The discharge capacities at 0.1 C are 161.1 and 159.6 mAh g^−1^ for pristine NCM and NCMS, respectively. During the initial charge to 4.3 V vs. Li/Li^+^, a gentle slope below 3.9 V occurs with the removal of lithium from NCM, which accompanies the oxidation of Ni^2+^/Ni^4+^ and Co^3+^/Co^4+^ [15]. Although the presence of sulfate was confirmed in the structural characterizations of NCMS, the decrease in its discharge capacity is not significant. The coulombic efficiencies are 99.4% and 99.7% at 0.1 C, 89.7% and 89.0% at 2 C, and 82.7% and 79.6% at 5 C for pristine and NCMS, respectively. These results indicate that excessive sulfate in NCM has a minimal effect on the reversibility in the Ni and Co redox system. The dQ/dV plots of pristine NCM and NCMS at 0.1, 2, and 5 C were obtained to examine the reversibility and redox overvoltage of the cathode active materials (Figure 7b). Both samples at 0.1 C display the reduction and oxidation peaks of Ni^4+^/Ni^2+^ at 3.74 and 3.76 V, respectively. The reduction peaks gradually shift to lower potentials as the C-rate during the discharge increases. However, the difference in the position of reduction peaks is very small between pristine NCM and NCMS. The capacity retention of pristine NCM and NCMS cycled at 1 C showed superior cyclability over 98% after 50 cycles in both samples (see Figure S1). Therefore, considering that pristine NCM and NCMS have the same redox overvoltage and the cycle stability, the sulfate groups belonging to NCM would not seriously affect the reversibility of the cathode active materials.

## 4. Conclusions

In this work, pristine NCM and NCMS are synthesized by co-precipitation and the effect of excessive sulfate is investigated on their structure, morphology, and electrochemical properties. The presence of sulfate in NCMS is examined by XRD and XPS results. SEM results show that NCMS has a porous surface and more voids than pristine NCM, which may cause the structural instability and deteriorate the electrochemical performance. In charge-discharge tests at different C-rates, the discharge capacities of NCMS at each C-rate is slightly decreased compared to pristine NCM. In summary, the unavoidable presence of sulfate, which originates from the sulfuric acid leaching of spent LIBs, has a minimal effect on resynthesized NCM cathode active materials as long as their precursors are adequately washed.

## Data Availability

The data presented in this study are available on request from the corresponding author.

## Supplementary Materials

The following are available online https://www.mdpi.com/article/10.3390/ma14216672/s1, Figure S1: Capacity retention of pristine NCM and NCMS at 1 C.
