# Supplementary material for: The Effect of Excessive Sulfate in the Li-Ion Battery Leachate on the Properties of Resynthesized Li[Ni1/3Co1/3Mn1/3]O2
Source: Materials (Basel). 2021 Nov 5;14(21):6672. doi: 10.3390/ma14216672 (PMC8587202; doi:10.3390/ma14216672)
Supplement: Supplementary file 1 [file materials-14-06672-s001.zip › materials-1403667-supplementary.pdf]

materials

MDPI

Supplementary Material

The Effect of Excessive Sulfate in the Li-Ion Battery Leachate on the Properties of Resynthesized $Li[Ni_{1/3}Co_{1/3}Mn_{1/3}]O_2$

Jimin Lee [1], Sanghyuk Park [1], Mincheol Beak [1], Sang Ryul Park [2], Ah Reum Lee [2], Suk Hyun Byun [2], Junho Song [3,*], Jeong Soo Sohn [4,*] and Kyungjung Kwon [1,*]

1 Department of Energy and Mineral Resources Engineering, Sejong University, 209 Neungdong-ro, Gwangjin-gu, Seoul 05006, Korea; txoio7612@naver.com (J.L.); shpark@sejong.ac.kr (S.P.); doqmsalscjf@naver.com (M.B.)
2 SungEel HiTech Co., Ltd., 143-12, Gunsansandan-ro, Gunsan-si 54002, Jeollabuk-do, Korea; qkrtkdfuf68@sungeel.com (S.R.P.); ahreum@sungeel.com (A.R.L.); shbn14@sungeel.com (S.H.B.)
3 Korea Electronics Technology Institute, 25 Saenari-ro, Bundang-gu, Seongnam-si 13509, Gyeonggi-do, Korea
4 Mineral Resources Research Division, Resources Recovery Research Center, Korea Institute of Geoscience and Mineral Resources, 124, Gwahak-ro Yuseong-gu, Daejeon 34132, Korea
* Correspondence: junesong@keti.re.kr (J.S.); jss@kigam.re.kr (J.S.S.); kfromberk@gmail.com (K.K.); Tel.: +82-2-3408-3947 (K.K.)

Citation: Lee, J.; Park, S.; Beak, M.; Park, S. R.; Lee, A. R.; Byun, S. H.; Song, J.; Sohn, J.-S.; Kwon, K. The Effect of Excessive Sulfate in the Li-Ion Battery Leachate on the Properties of Resynthesized $Li[Ni_{1/3}Co_{1/3}Mn_{1/3}]O_2$. *Materials* **2021**, *14*, 6672. https://doi.org/10.3390/ma14216672

Academic Editor: Alberto Vertova

Received: 16 September 2021
Accepted: 3 November 2021
Published: 5 November 2021

Publisher's Note: MDPI stays neutral with regard to jurisdictional claims in published maps and institutional affiliations.

Copyright: © 2021 by the authors. Licensee MDPI, Basel, Switzerland. This article is an open access article distributed under the terms and conditions of the Creative Commons Attribution (CC BY) license (http://creativecommons.org/licenses/by/4.0/).

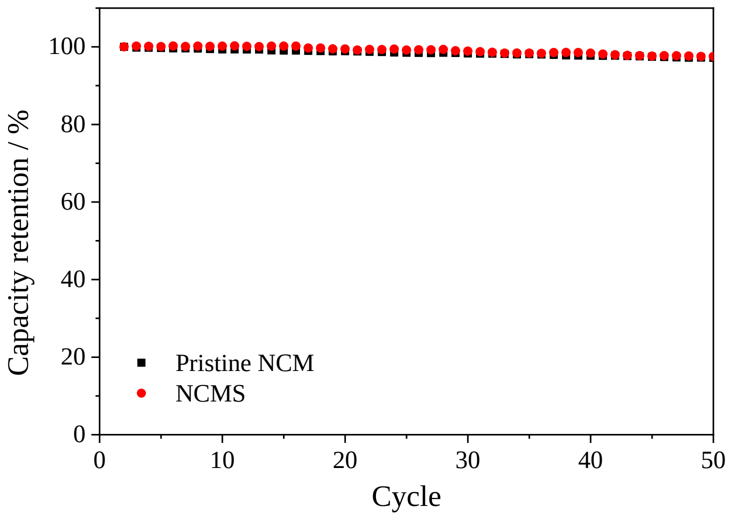

Figure S1. Capacity retention of pristine NCM and NCMS at 1 C.
